# Ubiquitous Mobile Knowledge Construction in Collaborative Learning Environments

**DOI:** 10.3390/s120606995

**Published:** 2012-05-25

**Authors:** Nelson Baloian, Gustavo Zurita

**Affiliations:** 1 Department of Computer Sciences, Universidad de Chile, Blanco Encalada 2120, Santiago, Chile; 2 Management and Information System Department, Universidad de Chile, Diagonal Paraguay 257, Santiago, Chile; E-Mail: gzurita@fen.uchile.cl

**Keywords:** mobile, collaborative learning, knowledge creation

## Abstract

Knowledge management is a critical activity for any organization. It has been said to be a differentiating factor and an important source of competitiveness if this knowledge is constructed and shared among its members, thus creating a learning organization. Knowledge construction is critical for any collaborative organizational learning environment. Nowadays workers must perform knowledge creation tasks while in motion, not just in static physical locations; therefore it is also required that knowledge construction activities be performed in ubiquitous scenarios, and supported by mobile and pervasive computational systems. These knowledge creation systems should help people in or outside organizations convert their tacit knowledge into explicit knowledge, thus supporting the knowledge construction process. Therefore in our understanding, we consider highly relevant that undergraduate university students learn about the knowledge construction process supported by mobile and ubiquitous computing. This has been a little explored issue in this field. This paper presents the design, implementation, and an evaluation of a system called MCKC for Mobile Collaborative Knowledge Construction, supporting collaborative face-to-face tacit knowledge construction and sharing in ubiquitous scenarios. The MCKC system can be used by undergraduate students to learn how to construct knowledge, allowing them anytime and anywhere to create, make explicit and share their knowledge with their co-learners, using visual metaphors, gestures and sketches to implement the human-computer interface of mobile devices (PDAs).

## 1. Introduction

Knowledge management (KM) theories identify two types of knowledge: explicit and tacit. Explicit knowledge is systematized and standardized, and can be expressed by a formal language. Tacit knowledge is acquired by people through verbal face-to-face conversations, exchange of personal experiences or by their own intelligence. Tacit knowledge resides in someone's mind and it is difficult to externalize because it is not structured enough [1,2]; it needs a certain social context in order to be interpreted [3]; or it is difficult to represent [4]. Nonaka and Takeuchi [5] proposed the socialization, externalization, combination, internalization (SECI) model in order to convert tacit knowledge into explicit in the so called knowledge construction (KC) process. As opposed to explicit knowledge, it might be very difficult to share tacit knowledge. Nevertheless, knowledge construction is an important factor in collaborative learning [6], especially from the constructivist learning theory point of view [7]. Explicit and tacit knowledge transfer may be of critical importance in a learning organization [8–10].

Much of the literature about KM systems addresses issues such as how to facilitate the creation, storage, and transfer of explicit knowledge [11]. By their nature such systems mainly handle explicit, codified knowledge, and there is little guidance on how to render tacit knowledge into explicit, so that it can be handled by the system. Researchers have mainly directed their research towards understanding the codification of explicit knowledge for KM and KC systems solutions, while the importance of tacit knowledge has gone unnoticed from an empirical perspective [5]. Most technologies used in KC range from storing best practices in databases to artificial intelligence systems supporting human decision making processes. These solutions have been so far designed for static workplaces and consequently require the corresponding infrastructure. This implies that mobile learners cannot be supported by the knowledge pool available in their organizations while performing their tasks outside the office, nor can they contribute to the knowledge pool at places and moments when they really can, *i.e.*, in ubiquitous environments [1,10,12,13].

Mobile workers often have spontaneous face-to-face meeting anywhere and anytime which are nowadays considered critical activities in any organization. In these meetings they develop processes involving social interaction, communication and mutual trust that yield to explicit and tacit knowledge creation [2]. It has been shown that mobile computing devices like PDAs, tablet-PCs, smartphones, *etc.*, are suitable and helpful for supporting and facilitating various communication and interaction processes among users requiring to perform face-to-face meetings and/or requiring computational support when being on the move in order to give continuity to their work when moving from one place to another [10,12]. The mobility and portability characteristics of these mobile devices, as well as their interconnection capabilities using wireless networks, allow their users to be connected in any moment at any place, which is vital for developing ubiquitous computing environments. On the other hand, the use of visual mechanisms to display and exchange information in mobile devices is supported by the fact that smart visualization mechanisms have been recognized to be powerful tools supporting knowledge communication, thus promoting knowledge sharing and knowledge construction [4].

Learning how to create and share knowledge by converting tacit into explicit knowledge, is not an easy task although it is part of the learning program of various courses associated to business school curricula, given the importance of this topic in relation to management of organizations and the value of the intellectual capital of its members. To our knowledge, the pedagogical practices applied so far are varied (case analysis, role playing activities, *etc.*) but there is still no evidence in the literature, nor curricula description, of pedagogical practices supporting the learning of the mentioned topic in an ubiquitous learning scenario, nor the usage of mobile devices in these situations.

Therefore, the aim of this work is build a system based on mobile technology (software applications running on PDAs) as technological support for this kind of ubiquitous meetings, providing simple but helpful visual mechanisms to support knowledge construction, especially targeted for tacit to explicit knowledge construction [1]. The resulting system called MCKC for Mobile Collaborative Knowledge Construction was evaluated by three pilot experiments in order to check the hypothesis whether MCKC effectively supports groups of users socially interacting among themselves in face-to-face scenarios while constructing and converting tacit to explicit knowledge by next design principles of the system: *functionalities that facilitate information contextualization, face-to-face social interaction, functionalities supporting brainwriting/brainsketching, selection of relevant information; visual presentation of the knowledge being created*.

The system's design takes into consideration empirical and experimental researches on the KC process and the tacit and explicit knowledge management, including: (a) recommendations about the role of information visualization mechanisms and using gestures and sketches as the main human-computer interaction paradigm with mobile devices having touch-screen in order to easily interact with the system and raise the productivity (Section 2.1); (b) results of researchers about the loss of productivity in the generation of ideas, like free-riding, production blocking, and evaluation apprehension, (Section 2.2); (c) the SECI knowledge transformation model [4,9,14] (Sections 2.3 and 2.4; and (d) recommendations about the role of mobile devices wirelessly interconnected supporting face-to-face social interactions [15,16] in knowledge construction processes when ubiquitous learning is required (Section 2.5). A conceptual model and the functional design principles applied in the design of MCKC system in order to support construction and tacit to explicit conversion of knowledge is presented in Section 3. A description of MCKC system is detailed in Section 4, its evaluation in Section 5, to finalizing with the conclusions in Section 6.

## 2. Knowledge Sharing and Knowledge Construction in Face-to-Face Scenarios

### 2.1. Visual Mechanisms, Sketching and Brain-Sketching

According to [4], systems using visualization mechanisms to manage information facilitate the sharing and construction of knowledge. In KC visualization is used to support the construction of tacit knowledge individually or collaboratively using sketches, concept maps, graphical representations, *etc.* It facilitates the clarification and enrichment of the tacit knowledge for the individual herself and when sharing her knowledge with others, supporting the development of different points of view.

Previous works on the field (e.g., [4]) highlight the following advantages of sketching in idea face-to-face generation meetings: (a) in relation to thinking, sketching stimulates a re-interpretative cycle in the individual participant's idea generation process, (b) in relation to communication, sketching stimulates the participants to re-interpret each other's ideas; and (c) in relation to the storing, sketching stimulates the use of earlier ideas in the idea generation process by enhancing their accessibility. The visualization technique called ‘brainsketching’ was used to describe idea generation techniques that use sketching. Thus being a graphic variation of the more widely known brainwriting technique. Van der Lugt [17] concludes that: (a) in idea generation meetings, sketches can stimulate creativity, especially in the immediate individual idea generation process; (b) sketches provide a more integrated group process by providing better access to the earlier ideas.

### 2.2. Brainwriting-Based Knowledge Construction in Groups

Most people believe that knowledge construction is best performed in face-to-face groups [18,19]. However, controlled research has shown that people produce fewer and lower quality ideas working in a group as compared with when working alone. Following causes for productivity loss in face-to-face brainstorming groups have been detected (a) free riding, to let to others group members do the work; (b) evaluation apprehension is when group members maintain a low production rate along the meeting session; (c) production blocking arises when group members cannot express their ideas as soon as they are generated. In accordance with [19], electronic brainwriting and brainsketching can be used to reduce or even eliminate these problems. Moreover, findings by [19] indicate that sharing written ideas in groups enhances creativity.

### 2.3. Knowledge Construction and Collaboration

Knowledge construction is a social, collaborative, and dynamic process transforming tacit into explicit knowledge [14,20–22]. It is produced by each person in the organization while doing their work individually or collaboratively in a face-to-face way.

**Figure 1.**
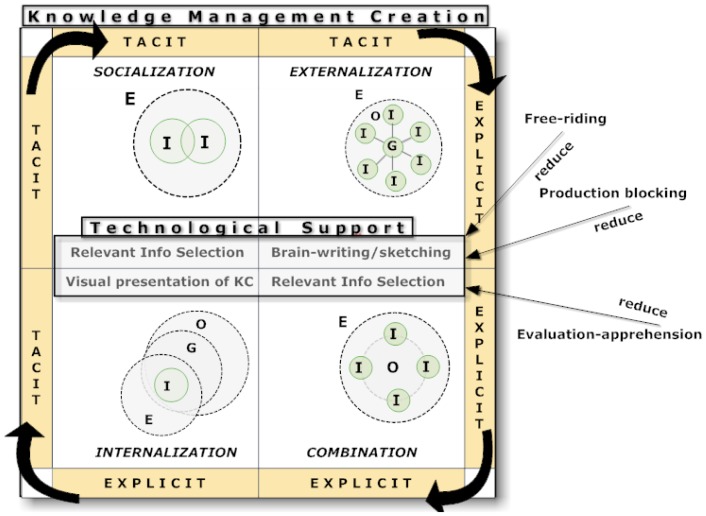
Conceptual model of MCKC based on SECI. E = environment, O = organization, I = individual, G = group.

Nonaka's SECI model [5,23], includes four ways of knowledge transformation (see Figure 1): (1) Socialization (tacit-tacit) is the assimilation process of tacit knowledge and its conversion to a new tacit knowledge among individuals who experience face-to-face collaboration. Here, knowledge is transferred by demonstration, observation, apprenticing, behavior modeling, and actual practice. (2) Externalization (tacit-explicit) is the conversion of tacit knowledge into explicit knowledge, which occurs when tacit knowledge is described or abstracted as concepts, formulas, rules and theorems. This process occurs within groups and communities. Knowledge is transferred through (partially) explicit information by the use of metaphors, analogies, prototypes or sketches. (3) Combination (explicit-explicit) is the production of new explicit knowledge through analyzing, classifying and sharing of explicit knowledge. In this case, knowledge is transferred formally and informally, by verbal or written means. (4) Internalization (explicit-tacit) refers to individuals or organizations applying theory to practice, turning explicit knowledge into one's own tacit knowledge through practice. Knowledge is transferred through common or shared understanding of abstract expressions or expert source of information.

Nonaka and Toyama [24], developed a KC model which involves a continuous interaction between tacit and explicit knowledge in order to produce new knowledge within groups or communities.

### 2.4. Applications of the SECI Model

The SECI model of knowledge creation was applied in two operational models for facilitating the dynamic creation of appropriate organizational knowledge [23]: middle-up-down management to leadership for parallel process which is suitable for promoting the efficient creation of knowledge in business organizations; and the hypertext organization that provides a structural base for the process of organizational knowledge creation.

Researchers in [23,25] report on SECI type systems used in organizations which trigger successful product innovation. Work by [16] has sought to empirically test the roles of knowledge assets in the promotion of SECI outcomes (socialization, externalization, combination, internalization), finding some support for hypotheses that assert that the presence of knowledge assets like organizational routines can have a strong impact on certain SECI outcomes.

Rice [26] applied the SECI model to multi-organizational projects, In accordance to them, applying the key elements of the SECI model across organizational boundaries, and thinking in creative ways as to how the implementation of SECI principles across organizations will create benefits for multi-organizational endeavors, will provide a potentially rich area of research and managerial development in future.

Naeve *et al.* [27], review the SECI knowledge creation theory of Nonaka and combine it with process modeling to arrive at a SECI process framework for the study and analysis of knowledge-creating learning processes, showing that the different SECI modes of knowledge conversion are empirically supported by pedagogical research. Naeve *et al.* [27], presented empirical pedagogical research that indicates how to effectively and efficiently apply the SECI knowledge creation process by connecting it to several important psychological and social motivators for learning. Naturally, both knowledge-transmitting and knowledge-creating learning processes have to be supported in workplace learning. As we see from the literature reviews the SECI model has been applied mostly in business scenarios to create and share knowledge thus supporting the “learning organization”.

### 2.5. Mobile Computing to Support Knowledge Construction and Ubiquitous Learning

Becerra-Fernandez *et al.* [10], state that knowledge is increasingly being acquired and shared on the move by learners working jointly in a face-to-face setting. According to [12] for the time being, the potential of KM is usually limited to static workplaces because most KM support systems are designed for being used in desktop PCs connected to a central server. This excludes a multiplicity of mobile learners. The authors in [12] argue that mobile KM supporting situated learning has not attracted as much attention as it should, considering its potential. There are mobile systems developed with the aim of extending the information access everywhere and anytime by using PDAs, [13]. Balfanz *et al.* [12] argue that mobile KM systems should be aware of the user's working situation stressing that system goals and KM methodology should be well focused.

In accordance to [28], ubiquitous knowledge construction is a vision for inspiring the development future learning scenarios. Mobile learners are the center of knowledge construction. Ubiquitous computing technologies, including mobile devices, wireless networks, and other advanced technologies, are tools required to implement the infrastructure for ubiquitous learning. By studying theories of constructivism, educators can better appreciate useful methods facilitating learning at the right time at the right place. Constructivism expands the width of ubiquity by taking into account learners' motivation, articulation, collaboration, social interaction and reflection in the context of meaningful learning [29,30]. The ubiquitous availability of mobile devices promotes the seamless learning notion that envisages the embodiment of learning into everyday living. The notion of seamless learning advocates, “learning anytime, anywhere” and not “learning every time, everywhere.” [31]. As mentioned in [31], we also do not mean that seamless learners are always doing tasks and pursuing learning especially outside of school. Rather, the goal is to empower and support them to learn wherever and whenever they are stimulated to learn, and not to require them to learn every single second they are awake.

## 3. A Conceptual Model and Functional Design Principles to Support KC

In [32] its authors conclude that rather than focusing on systems to codify knowledge, we should instead concentrate on systems that facilitate collaboration between knowledge holders, creators and those needing the knowledge and some authors recognize the need for face-to-face KC and knowledge sharing support in order to facilitate the transfer of complex, context-specific knowledge [33].

Balmisse *et al.* [34] name key functional requirements for KM systems: (1) facilitate information contextualization—better results are often associated with access to the conceptual representation and the structure of information, (2) facilitate social interactions and networking—digital socialization systems need to encourage spontaneous as well as casual meetings anywhere and at any time with multiple views or modes of interactions, and (3) present a ease to use human-computer interface. We adopt these requirements as design principles for our proposed system.

Considering the arguments and ideas mentioned before, we decided to develop a prototype of a KM system supporting KC in mobile scenarios with people collaboratively working face-to-face, called MCKC, Mobile Collaborative Knowledge Construction. Its design principles are derived from the various results of previous empirical and experimental KC research works and human-computer interface design principles.

MCKC runs on PDAs wirelessly interconnected by an *ad hoc* network. It also offers the possibility of synchronizing data on the mobile devices with a central repository when the required networking infrastructure is available, allowing people have access to the existing knowledge anytime and anywhere. The touch-screen mechanism of their displays is used as the main human-computer interaction mean to input information to the system. MCKC implements the free-hand-based command and data input paradigm which means users are able to draw sketches, edit graphic information and enter free-hand written text, as well as using visual metaphors for information management, like conceptual maps consisting of information nodes and relations among them. They can also associate each of the information nodes they create with various documents, like text files, images, *etc*.

The system's design is oriented to facilitate KC collaboratively based on the SECI model. According to this, the system has three modes: (1) *brainwriting/brainsketching*, or knowledge externalization support mode, (2) *selection of relevant information*, or socialization and combination support mode, and (3) *visual presentation and knowledge semantics of the created knowledge* mode, which is associated to the socialization process. Each mode responds to different requirements. The MCKC conceptual model shown in Figure 1 presents these three modes as different technological support mechanisms to the four relevant KC processes proposed by the SECI model [5].

In order to reduce the productivity problems associated to generation of ideas, during the brainsketching/brainwriting mode each user can externalize her ideas despite there are other colleagues doing the same. MCKC allows each person to use her own device to develop her knowledge, without having to pay attention to other being created by other users at the same time. They can explain their ideas to others in the next step.

The three modes of the system are aimed to provide the environment that facilitates tacit to tacit knowledge sharing and construction, without the need to convert tacit knowledge to explicit knowledge before sharing it. There are three major aspects that contribute to make the conversion of tacit knowledge to explicit knowledge difficult: (1) people are unaware of the tacit dimension of their knowledge, (2) is not necessary for an individual to make tacit knowledge explicit in order to use it, and (3) the risk of losing power within the organization by sharing the knowledge. Hence, face-to-face communication should be used to share and personalize tacit knowledge, rather than extract and store it. The exchange of tacit knowledge is viewed as a social process between people that requires face-to-face interaction.

MCKC considers social interaction as a key factor for collaborative KC, although it can be also used to support individual KC. As shown in Figure 1 in this way we expect to reduce the productivity problem described before. Writing ideas instead of speaking them inside a group minimizes the problem of production blocking since individuals do not have to wait their turn to generate ideas. It may also reduce evaluation apprehension since the written format eliminates the need for public speaking and is typically more anonymous than oral brainstorming. Also the free-riding problem might be reduced because it will be easier to identify not contributing people.

## 4. MCKC System Description

Sketches help to externalize tacit knowledge and hence express ideas and concepts which people have in their minds. They can also help people to order and clarify their own ideas before communicating them to others [17,35]. The MCKC interface allows the manipulation of information in a simple way using the device's stick to activate options using gestures. It uses visual mechanisms for presenting and manipulating information. It allows the edition of sketches and freehand writing and facilitates the interaction among members of a group working face-to-face collaboratively. MCKC can be used anytime, anywhere, which means it can be brought to any physical place of the organization, and can be used while people are on the move. In this section, we describe briefly the functionalities of MCKC, its three working modes and the characteristics that make it a suitable tool for supporting KC. Each mode is oriented to support one stage of the SECI model. The system does not impose a certain order of sequence for using each mode. It is always possible to go back to a previous mode in order to make corrections or even start from scratches again.

This description is based on a learning scenario experience performed to evaluate the system, where students' task was to develop a marketing strategy proposal for a certain product based on information and specific features about a product. They had to develop ideas about how a new advertising poster for an all-terrain car should look like. The screenshot presented in this section are result of this experience. The results of the evaluation are presented in the next section.

**Figure 2.**
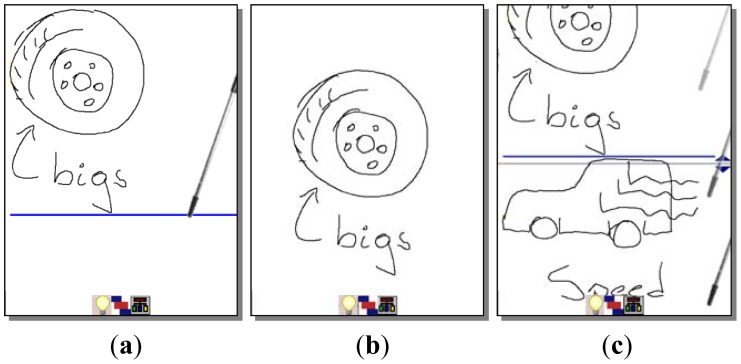
Ideas specification through sketching and freehand writing. (**a**) An idea consisting in a wheel and the text “big s” is generated; (**b**) Writing a horizontal line through the whole screen will mark the separation of two ideas; In (**c**) we see how the second idea is being produced with a sketch of a car and the “speed” text.**c.**

Two versions of the MCKC system were implemented in C# for PDAs and Tablet-PCs with the same functionalities and the application adapts the workspace to the screen's size. In small screens the system's workspaces will appear with scrolling bars in order to match the size of the bigger screens. When a group of users start MCKC an *ad hoc* network between their devices is automatically established. The first view of the interface is a white workspace with icons on the bottom to select one of the three modes the system supports (see bottom of Figure 2(a)).

**Figure 3.**
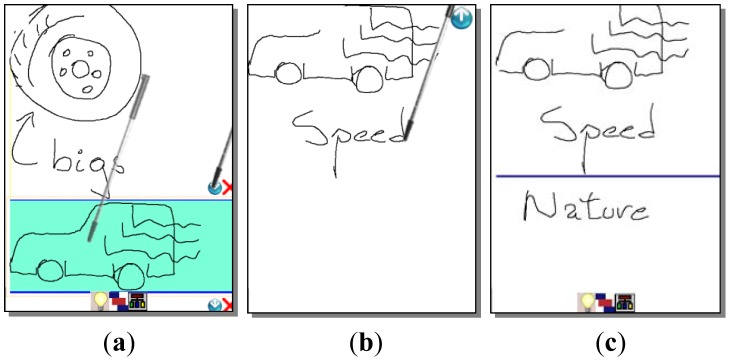
Editing an idea. (**a**) An idea is being selected and then the “enter” icon is clicked; (**b**) The idea is being edited; (**c**) Updated idea after leaving the editing mode

The brainwriting/brainsketching mode supports knowledge externalization allowing users to explain their tacit or explicit knowledge by means of freehand writing or sketching. This mode works in a non-collaborative way by default, allowing users to prepare their ideas before sharing them, reducing the free-riding, production blocking, and evaluation apprehension problems. Users generate their ideas in parallel during a face-to-face session (see Figure 2). If a previous idea has to be edited, the user selects the area where it is by a single click and “enters” the edition mode clicking the “arrow down” icon (see Figure 3(a)).

### 4.1. SECI—Externalization: Brain-Writing/Sketching Mode

The system offers an option to start working collaboratively. Users share their ideas and start editing them collaboratively in the same way they did individually. This supports the knowledge socialization process of the SECI model. Since ideas are shown one below the other a scrolling function is necessary to go through them, which is done by a gesture of sliding the stick up and down parallel to the right vertical border of the screen (see Figure 2(c)).

### 4.2. SECI-Socialization/Combination: Relevant Information Selection Mode

**Figure 4.**
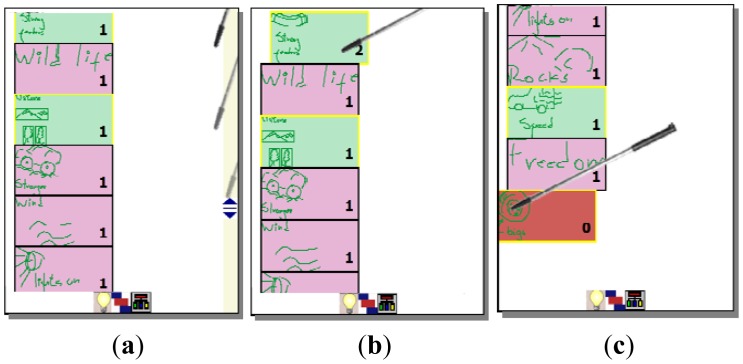
List of ideas to be selected. (**a**) Scrolling the ideas; (**b**) An idea being ranked to the level 2; (**c**) An idea ranked to the 0 level.

After each user has externalized her ideas individually or collaboratively, it is necessary to refine them collaboratively in order to define which are relevant and which not. In order to support this process, MCKC generates a list of all created ideas, which is shown as similar rectangular boxes with colors associated to the author. At this stage, the list of ideas is visible to all participants, as shown in Figure 3. In order to rank them, participants have to vote for them positively or negatively. They can issue a positive vote for a certain idea by making a tick gesture on the left area of the rectangle representing it (see Figure 4(b)), or negative one making a tick on the right area of the rectangle (see Figure 4(c)). Numbers from 0 to 5 represent the ranking of each idea according to the votes received, being 5 the most relevant. A scroll mechanism is also available in this mode (Figure 4(a)).

**Figure 5.**
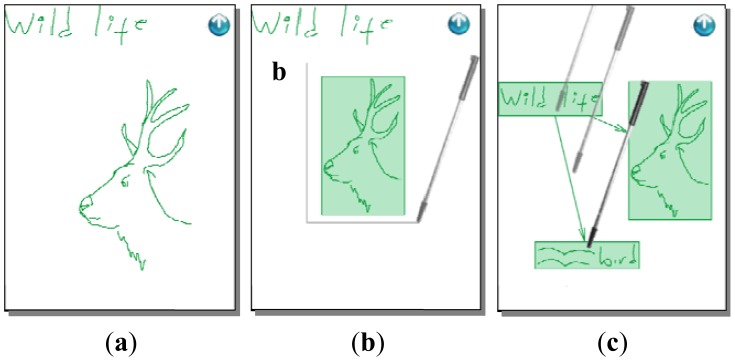
Collaborative idea. (**a**) An idea's content in the editing mode consisting of a sketch and freehand written text; (**b**) The sketch being converted into a node; (**c**) Relating nodes by creating arrows linking them.

Ideas start with a ranking number equal to 1. The ranking number appears in the bottom-right corner. As ideas get ranked, they will be rearranged according to the ranking. In this way, relevant ideas are easily differentiated facilitating their selection. It is possible to add more information to an idea by including concept maps. Concept map's nodes can be created inside an idea by making an “L” gesture enclosing a single or a group of strokes previously drawn (see Figure 5(a)). This will be the node's label (Figure 5(b)). Users can get inside of these nodes to include more information as well as to create more nodes, creating a hierarchy of nodes. Nodes in the same level can be related to each other by drawing a line connecting two of them. This line will be converted to an arrow which will remain linking both nodes even if they are moved (Figure 5(c)). These conceptual maps allow users to organize and add a semantic meaning to the information. In order to have an overview of the concept maps already created and also in order to easily access them, MCKC offers a tree-view of them.

**Figure 6.**
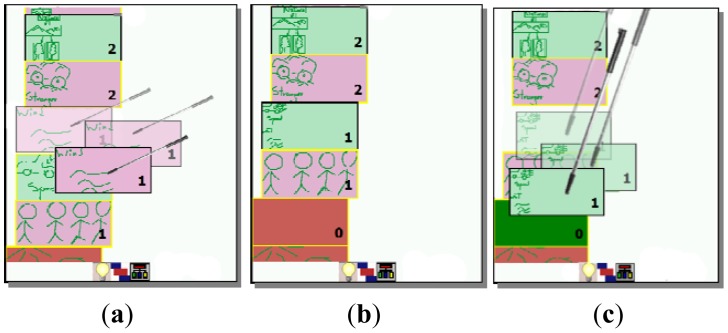
Ideas merging. (**a**) Two ideas merged; (**b**) Rectangle containing the merged idea is situated at level 0; (**c**) The undo action.

It is possible to merge ideas that might be similar. For this, the user can drag the rectangle representing one of them (Figure 6(a)) and drop it over the idea which is similar (Figure 6(b)). An empty red rectangle at the end of the ideas' list will appear representing the idea which was moved. This is done in order to allow the “undo” of this action, by dragging the rectangle back and dropping it over the empty red rectangle (Figure 6(c)).

### 4.3. SECI-Internalization: Visual Presentation and Semantic of the KC Mode

**Figure 7.**
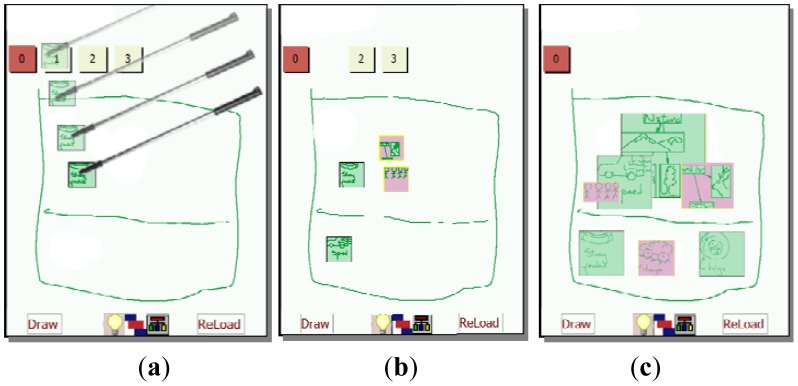
Visual representation of the proposed ideas. (**a**) An idea is selected, dragged and dropped; (**b**) All ideas of level 1 were use on the KC representation, and this set disappears; (**c**) Final result.

This mode allows users to concretize the KC process using a final visual representation of the ideas. This process is done collaboratively with the agreement of all participants. At the beginning of this mode, an empty page will appear with a list of small squares at the top representing the ideas generated ordered according to their ranking. In this stage, participants have to make a visual arrangement of the ideas. This is an important stage during the KC since tacit and explicit knowledge is expressed by means of sketches and other visual representations. It is expected that participants will first draw a sketch over which ideas will be placed in a particular order according to the meaning of the sketch. In the example shown in Figure 7, users determine the position where each idea will be placed in a poster. Ideas can be dragged from the list and dropped in the desired place (Figure 7). The placement of the ideas inside the sketch should represent a meaning collaboratively defined by all participants. The square representing an idea can be resized as desired (see Figure 7(c)). After placing the ideas on the schema, participants may finalize their proposal or they can go back to a previous mode in order to edit, the existing ideas or include new ones. Not used ideas might be deleted.

## 5. MCKC System Evaluation

Antunes *et al.* [36] describe several proposed methods to evaluate collaborative applications, which comprise a variety of approaches with various goals. Three evaluation scenarios, called role-based, rule-based and knowledge-based were proposed jointly with a set of guidelines to select the appropriate evaluation methods. Antunes *et al.* [36] propose a guideline for selecting the most adequate evaluation method for a collaborative system, which is related to the development status of the software application being assessed. Since in our case the application has already been developed, we apply a knowledge-based scenario, in order to find out if the system functionality matches the goals and purposes. Furthermore, the knowledge-based scenario is the most adequate to software applications or systems supporting interaction, collaboration and decision-making. In the knowledge-based scenario, the evaluation is mostly focused on the all members' impact. The evaluation methods employed in this scenario are: (a) cooperation-scenarios, (b) scenario-based evaluation, (c) perceived value and (d) “quick and dirty” ethnography. Our analysis of the evaluation methods based on the description given in [36], the characteristics of the system we are going to evaluate and the type of final users of the system allows us to conclude that the “scenario-based evaluation” is the most suitable.

### 5.1. Application of the Scenario-Based Evaluation Method

#### 5.1.1. Hypothesis and Data Collection Method

In the scenario-based evaluation method [36], the evaluators perform semi-structured interviews with end-users and claims about them. Then, focus groups validate these findings. The frequency of claims helps to quantify the contribution of the system perceived by the end-users. By applying these data collection methods we check the hypothesis whether MCKC effectively supports collaborative knowledge creation and the conversion of tacit into explicit knowledge due to the face-to-face social interaction among the group members involved in the task. In order to answer this question we evaluate if the design principles of MCKC, which were described and analyzed in the conceptual model in Section 3 were fulfilled by the resulting application. These principles are: (a) functionalities that facilitates *information contextualization*, facilitates *face-to-face social interaction*, and implements *an ease to use human-computer interface*, which are according to the literature these first three principles promote knowledge creation; (b) functionalities supporting *brainwriting/brainsketching*, *selection of relevant information*, *visual presentation of the knowledge being created*, which are principles proposed by the model of conversion of tacit to explicit knowledge; and finally, (c) r*educing the productivity loss of groups due to free riding, evaluation apprehension, and production blocking*, identified as an aspect the system should help to reduce by anonymously registering the contributions of the participants.

#### 5.1.2. Final Users, Tasks Performed, Equipment Used, and Experimental and Control Groups

The system was evaluated performing three pilot experiments in real ubiquitous working scenarios involving 18, 12 and 12 test users respectively. The experiments were performed independently of each other with different students. All test user samples were last-year undergraduate students from the School of Business and Economics, of the Universidad de Chile with an average age of 24.6.

The first experimental scenario consisted of two groups, totaling 18 students, nine were randomly assigned to the experimental group, and nine to the control group. All of them had already passed the marketing course foreseen in their studies program. Their task was to develop a marketing strategy proposal for a certain product based on information and specific features about a product that has to be released to the marked. For the second and third experimental scenarios two students groups with 12 students each were engaged, six students were randomly assigned to the experimental group, and six to the control group. All of them had already passed the IT and Business course foreseen in their studies program. Their task was to identify a real case for which the introduction of IT would be an improvement. The aim of these activities was to explore how each group of students conducted the necessary KC in order to achieve the proposed goals. In all pilot experimental scenarios, the experimental groups used the MCKC system to support their meetings and the control group was allowed to use pen and pencil and standard “of the shelf” software like word processors, spread sheets, design and drawing software, in their notebooks without touch screen and not using any general application synchronizing software. Students were provided with Compaq Ipaq H3970 PDAs equipped with Windows Pocket PC 2002 operative system, Wi-Fi connectivity, in order to establish an *ad hoc* network every time a working meeting was required, a 3.5 inch screen and batteries with the double capacity than the originals in order to achieve a continuous functioning time of at least four hours, which was enough time to perform two meetings a day. Although there are more powerful devices nowadays, the PDAs were powerful enough to run MCKC and it was possible to use a stylus as input device, which facilities the creation of sketches, handwriting and activation of gestures.

#### 5.1.3. Research Procedure

The research procedure was as follows: First, all experimental and control groups had a session where the task was explained in about 15 minutes. The experimental groups had another session in which they learned how to operate MCKC. This session was 45 minutes long.

After the introductory sessions all experimental and control groups of the tree pilot experiments were instructed to have ubiquitous collaborative meetings at anytime and anywhere at the School dependencies in order to propose, discuss and develop ideas for a marketing strategy. Meetings were arranged opportunistically among them or other faculty students and especially with any lecturer they considered relevant to ask for help or to express an opinion in their own offices or right after lectures. The only constrain was that they should have at least six meetings during a period of time of two weeks. So, there should be in average three sessions per week; each session should be from 0.5 to 2 hours long. After this activity the students had to submit a final electronic document describing their ideas for a marketing strategy of the product or a problem with a valuable IT solution. The analysis of the marketing strategy proposal and an added value IT solution tasks were conducted by three marketing, and IT Business experts faculty members respectively and evaluated with marks ranging from 1 (worst) to 7 (best).

One day after the last session of each experimental scenario, we asked each student of experimental and control groups to individually complete a semi-structured interview about most positive and negative aspects of the system. The questions of the semi-structured interview were aimed at identifying the impact of the system on the design principles of MCKC described at the beginning of this section. A week after, during which the answers of the semi-structured interview were analyzed, and their frequency registered on a table, six focus groups sessions were performed, one for each performed task, experimental and control group. The focus groups were aimed at validating the results collected during the interviews. After each session, the processes required for knowledge creation and the conversion of tacit into explicit knowledge described in Section 2 were explained to the students and they were asked if they did performed these processes during the performance of the tasks they were assigned. This allowed us to compare to which extend the evaluated system supported the students in performing these processes.

### 5.2. Evaluation Results

Although all six groups performed the minimum required number of sessions control groups performed less meetings (seven, eight and six, respectively) than the experimental groups (nine, 10 and eight). This was mainly attributed to the fact that opportunistic working sessions were easier to initiate using PDAs, contrary to the control groups using standard software, which had to prepare meetings in advance.

On the other hand, information generated and used during meetings with MCKC was easily accessible for the next meeting, contrary to the paper sheets which were managed by only some of the control group members which were not easily shared during a meeting.

#### 5.2.1. Task Results

Regarding the tasks we got following results: for the marketing proposal all three faculty members expressed that the quality and completeness of the proposal submitted by the experimental group were better; however the proposal of the control group was fairly good. The experimental group work surpassed the quality of the control group work in identifying more precisely the goal market. Also their strategic plans were more clearly described. The evaluation mark for the experimental group was 6.5 and for the control group 5.3. For the second experiment both the problematic identification and the IT solution proposed were developed with better precision and more solid basis by the experimental group. They received a 6.7 against a 5.5 of the control group. For the third group the marks were not significantly different with a 6.6 for the control group and a 6.1 for the experimental group.

#### 5.2.2. Results Obtained for the Semi-Structured Interviews

The answers to the semi-structured interviews were analyzed and tabulated using classification techniques, like dialectic reduction and triangulation, into five categories of the Likert scale: poor, deficient, fair, good, excellent, for the following design principles of MCKC: *functionalities that facilitate information contextualization, face-to-face social interaction, functionalities supporting brainwriting/brainsketching, selection of relevant information; visual presentation of the knowledge being created*. For design principles *using an easy to use human-computer interface* and *reducing the productivity loss of groups due to free riding, evaluation apprehension, and production blocking* results could not be quantitatively tabulated since they corresponded to qualitative descriptions and were less frequently mentioned than the other principles. Results for these design principles will be explained later. The results for the first mentioned design principles are shown in Table 1.

In order to have a summary number which reflects how good the students considered the computational support they had en each case for carrying on their task we converted the values of the Likert scale in numbers (P = 1, D = 2, F = 3, G = 4 and E = 5) and we computed a mean value for each aspect for each group. Table 2 shows the results. According to them, on average MCKC introduces improvements in all aspects. Individually, we see that for the third group, the control group rated the standard computational support better than the experimental group for design principles *face-to-face social interaction* and *selection of relevant information*. Interestingly, in this group the quality of the resulting work was not different between the experimental and control groups. Coherently, we find the biggest difference between the average evaluation of the control and experimental group for the first experiment (2.58 against 3.60), where there is also the biggest work quality.

During the semi-structured interviews the students of the experimental group highlighted the easiness to synchronize data and information input as well as the awareness about who is presenting and/or explaining something to the rest. Second, they said MCKC did not hinder the face-to-face contact, which was also important to achieve their goals. Third, the visualization of the relevant information and the possibility of organizing their ideas under a visual logic was the second most appreciated aspect of the tool. The results of the experiment, as well as the interviews conducted with both groups show that, with high probability, MCKC has a positive impact in KC.

Furthermore, the semi-structured interviews revealed that the most difficult aspects to perform for the control groups were the facilitation of information context, and brainstorming/brainsketching processes due to the difficulty to synchronize their work, especially for the group that used pen and paper for making sketches before they prepared the electronic document to submit. Also selection of relevant ideas was reported difficult to perform with standard computer support, especially when the number of participants in the session increased.

With respect to the design principle *using an easy to use human-computer interface*, we found that the human-computer interface highly contributed to an easier sharing and synchronization of data representing their ideas. This last opinion of members from the experimental group highly contrasts with the opinion of one member of the control group who expressed that sharing the working documents was one of the main difficulties. The use of the three modes of MCKC was considered very useful. Especially appreciated was the opportunity to exteriorize their ideas through sketches they can share immediately

#### 5.2.3. Results Obtained for the Focus-Group

The focus groups with all students of the experimental scenarios revealed that the free-riding, production blocking, and evaluation apprehension problems were partially mitigated. The explicit knowledge could be easily specified and communicated with the help of MCKC. It was also noted that that sketches helped to exteriorize and share tacit knowledge. The visualization of the artifacts on the system interface associated to data, information and functionalities triggered by gestures was well accepted and easy to use. However, more experimented users missed the menus, choice boxes and fast access keys.

The design principle associated to *visual presentation and semantic of the created knowledge* mode, was perceived as very helpful one because it offers a flexible and rich way to represent knowledge as a final result of a goal. In the second place, the *brain-writing/sketching* mode was perceived as the most helpful mode. In the whole MCKC was perceived as a relevant tool to support collaborative work because it enables people to contribute, explain, exteriorize and share their ideas. Regarding the usability of the MCKC, in general participants suggested some additional improvements. Participants regarded a major challenge to keep the awareness information and collaboration constantly up-to-date. The learning curve of MCKC was satisfactory completed during the second working session. Some difficulties were perceived on users who declared not having too much experience with mobile and touch screen technologies.

Students participating in the control group mentioned that they faced more problems related to *productivity loss of groups due to free riding, evaluation apprehension, and production blocking*, than the students from the experimental group, where the participation of all members was higher and conveniently mediated by MCKC. The fact that MCKC users could develop their ideas simultaneously contributed to increase the participation. The anonymity of the contributions allowed for a more objective analysis of the proposed ideas.

Finally, during all the focus-groups sessions, after the design principles were explained to the students, the members of the experimental groups considered that MCKC supports knowledge creation processes and the conversion of tacit into explicit knowledge. It is important to mention that although students had some knowledge about the theory of knowledge construction they did not realized that the system they used was actually developed to support this process until the design principles of MCKC were explained to them during the focus group. Even after this explanation many students sustained that most of the knowledge creation work depends more on people's capacity than the computational mediation. Some members of the control group also realized only after the explanation that they were applying some of the knowledge creation principles; however they were not as convinced as their colleagues of the experimental group. They also mentioned that they felt the computer support they used did not really support this process.

## 4. Conclusions

Knowledge construction and tacit to explicit knowledge conversion are important activities in constructivist collaborative learning. People possess a large amount of tacit “hidden” knowledge which has to be converted into “new knowledge”, in order to promote its sharing and innovation. MCKC is a tool that helps externalize knowledge, especially tacit knowledge, among members of a learning group, as the last testing activity we conducted has shown. Our work is based on the empirical and experimental findings of knowledge creation related works, which have been incorporated into the system MCKC presented in this paper. The visualization technology of knowledge and the use of mobile devices as support for knowledge creation is a new field, which has already generated applications for different scenarios such as engineering, education and economy.

Our application supports the visualization of information in a free and extensible way. It also promotes the collaboration in ubiquitous scenarios by making use of *ad hoc* wireless networks, which helps to transform tacit into explicit knowledge, promoting the elicitation, transmission and sharing of information based on sketches. The knowledge management success model developed by [8] emphasizes the need for these systems to include both types of knowledge (tacit and explicit) and linkages or pointers to people with knowledge expertise. A better understanding of the various characteristics of the tacit knowledge dimension, as detailed in the present study, will assist researchers and practitioners in the development of more sophisticated knowledge management systems that can adequately address knowledge users' needs for both codified knowledge and interaction with human sources of knowledge.

As for the face-to-face channels that users establish and the trust level they might develop during the use of this system, we can say they are more dependent on the individuals themselves than on the support a system like this is able to provide. This means that the effectiveness of any knowledge management system will always depend on the abilities, attitudes and intentions of its users and the level of trust they are able (or willing) to develop. In the near future we will focus on improving many functionalities of the prototype, studying alternative interaction modes, trying to minimize the overhead necessary to maintain data validity. Also, we will conduct more extensive tests of the prototype improved.

## Figures and Tables

**Table 1.** Frequency of results obtained from transforming the qualitative results of the semi-structured interviews into a value on the Likert scale.

	**Experiment 1** **Task: Marketing strategy proposal**										**Experiment 2** **Task: Added value IT solution**										**Experiment 3** **Task: Added value IT solution**									
	**Control Sample: 9**					**Experimental Sample: 9**					**Control Sample: 6**					**Experimental Sample: 6**					**Control Sample: 6**					**Experimental Sample: 6**				
	P	D	F	G	E	P	D	F	G	E	P	D	F	G	E	P	D	F	G	E	P	D	F	G	E	P	D	F	G	E
**Facilitation of information context**	2	2	3	2		1		2	5	1	1	2	2	1			1	2	3			1	3	2			1	3	1	1
**Face-to-face social Interaction**		3	4	2			3	1	4	1		1	3	2				2	2	2		1	2	3			2	3	1	
**Brainwriting brainsketching**	1	2	4	2			1	2	3	3	1	1	3	1			2	3		1	1	2	2	1			1	1	2	2
**Selection of relevant information**		4	3	2			3		5	1	1	3	2				2	3	1		1	1	2	2		2		2	2	
**Visual representation of knowledge created**	3	4	2				1	2	4	2	1		3	2			1	2	1	2		3	2	1			2	3		1

P = Poor, D = Deficient, F = Fair, G = Good, E = Excellent

**Table 2.** Summary of the evaluations. Mean values each group gave to each one of the design principles when P = 1, D = 2, F = 3, G = 4 and E = 5.

	**Control Groups**				**Experimental Groups**			

	Exp.1	Exp.2	Exp.3	Avg.	Exp.1	Exp.2	Exp.3	Avg.

**Facilitation of information context**	2.56	2.50	3.17	2.74	3.55	3.33	3.33	3.40
**Face-to-face social Interaction**	2.89	3.12	3.33	3.11	3.33	4.00	2.83	3.39
**Brainwriting/brainsketching**	2.78	2.67	2.50	2.65	3.89	3.00	3.83	3.57
**Selection of relevant information**	2.78	2.12	2.83	2.58	3.44	2.83	2.67	2.98
**Visual representation of knowledge created**	1.89	3.00	2.67	2.52	3.78	3.67	3.00	3.48

**Average**	2.58	2.68	2.90	2.72	3.60	3.37	3.13	3.36
